# Mid-Term Outcomes After Open Reduction Internal Fixation of Proximal Interphalangeal Joint Dorsal Fracture-Dislocations Through a Volar, Shotgun Approach and a Review of the Literature

**DOI:** 10.2174/1874325001711011073

**Published:** 2017-09-30

**Authors:** Juan Marcelo Giugale, Juntian Wang, Robert A. Kaufmann, John R. Fowler

**Affiliations:** 1 Orthopaedic Surgery Resident Department of Orthopaedic Surgery University of Pittsburgh Medical Center Pittsburgh, PA, USA; 2 Medical Student University of Pittsburgh - School of Medicine Pittsburgh, PA, USA; 3 Associate Professor Department of Orthopaedic Surgery University of Pittsburgh Medical Center Pittsburgh, PA, USA; 4 Assistant Dean for Medical Student Research Assistant Professor Department of Orthopaedic Surgery University of Pittsburgh Medical Center Pittsburgh, PA, USA

**Keywords:** Proximal Interphalangeal (PIP) Fracture, PIP dislocation, Open reduction internal fixation, Shotgun Volar Approach, Long term outcome, Review of Treatment Options

## Abstract

**Background:**

Proximal interphalangeal (PIP) fracture dislocations remain a complex injury pattern to treat. There are several treatment methods available aimed to restore stability, preserve range of motion, and reconstitute the articular surface. This study looked at the mid-term clinical and radiographic results of open reduction internal fixation through a shotgun approach of comminuted PIP fracture dislocations.

**Methods:**

A retrospective review was conducted of all PIP fracture dislocations treated through a volar, shotgun approach at a single institution over a 15-year period. Patients identified were contacted and asked to return to the office for clinical and radiographic evaluation. Patient reported outcomes were assessed with the Michigan hand questionnaire (MHQ) and visual analog scale (VAS) for pain.

**Results:**

5 patients returned to the office for further evaluation with average follow-up of 69 months (range, 33-133 months). 3 patients were found to have post traumatic arthritis on radiographs. 1 case had recurrent instability and one case had a deep infection, both necessitating further surgical intervention. Average PIP arc of motion was found to be 79°. Average VAS score of 0 and MHQ result of 95 (out of a possible score of 100) indicating no residual pain and excellent functionality of the affected hand.

**Conclusion:**

Open reduction internal fixation of comminuted PIP fracture dislocations utilizing the volar, shotgun approach provides excellent mid-term functional results despite the high incidence of post traumatic arthritis.

## 1 INTRODUCTION

 Proximal interphalangeal joint (PIP) fracture dislocations continue to be a challenging fracture patterns to manage. If suboptimally treated, this injury complex can be potentially debilitating secondary to subsequent stiffness, pain, development of arthritis, and limited function of the affected digit.

Dorsal fracture dislocations are more common than volar dislocations and are considered unstable when the fracture fragment of the middle phalanx base is over 50% of the articular surface or more than 30° of flexion is required to maintain a reduced joint [1]. As described by Kiefhaber and Stern, the mantra of treatment involves restoring a congruent, stable joint throughout the arc of motion, restoration of the articular surface, and early range of motion [2].

A number of surgical options have been described for the treatment of unstable fractures including, external fixation, volar plate arthroplasty, hemihammate autograft, and open reduction internal fixation (ORIF) with mini screws, plates, and Kirschner wires. ORIF with mini-screws has been traditionally reserved for fractures with minimal comminution and articular impaction

Few studies have investigated the long term outcomes of ORIF treatment of dorsal fracture-dislocations of the PIP joint. This study reviews our experience with ORIF of PIP dorsal fracture-dislocations treated through a volar, shotgun approach with mini-screw fixation. The aim of this study is to determine the mid-term objective and subjective outcomes of patients who underwent this surgical procedure for PIP dorsal fracture-dislocations.

## 2 MATERIALS AND METHODS

After obtaining approval from the institutional review board, a search was conducted using CPT codes (26735: ORIF middle/proximal phalanx, 26747: open treatment articular fracture middle/proximal phalanx, and 26785: open treatment interphalangeal dislocation with or without external fixation) over a 15-year period (2000-2015).

Medical charts and radiographs were reviewed to identify patient demographics, injury complex, surgical procedure. The identified patients meeting inclusion criteria were asked to complete the Michigan Hand questionnaire, a validated instrument used to measure patient reported outcomes [3]. These patients were also asked to return to the office for radiographs looking for the development of arthritis and clinical exam to evaluate range of motion and grip strength. Frequency counts and percentages for nominal variables and measures of central tendency (means, medians) were determined.

All surgeries were performed at a single institution. The surgical technique performed is as follows. A volar Brunner incision was utilized in most cases. The radial and ulnar neurovascular bundles were identified and protected. An incision was made through the A3 pulley. The A2-A4 were preserved. The volar plate was identified and usually found to be attached to the middle phalanx base fracture fragment.

The check-rein ligaments were carefully released. The middle phalanx was then hyperextended, into a shotgun position, exposing the entire articular surface. Articular comminution and impaction was visualized. Fracture fragments were reduced, held with small caliber K-wires and fixed with mini-screws. Once, the articular surface was restored, the volar plate was repaired with 3-0 vicryl suture in horizontal mattress fashion.

## 3 RESULTS

12 patients with PIP dorsal fracture dislocations that underwent ORIF through a volar approach with mini-screw fixation were identified. These 12 patients were contacted and 5 patients returned to clinic for radiographic and clinical evaluation Average follow-up was 69.4 months (33-133 months). The demographics of these patients are seen in Table **1**.

4 out of 5 patients underwent surgical fixation within 7 days from injury. 1 patient attempted a short course of conservative treatment before undergoing surgical intervention 16 days after her injury.

4 out of the 5 injuries sustained their injury as the result of blunt trauma. One patient’s injury was the result of a circular saw. This injury complex, unlike the other four included patients, involved transection of the ulnar and radial neurovascular bundles, the flexor and extensor tendons. In addition to open reduction internal fixation through a modified volar approach (utilizing the traumatic laceration), revascularization and tendon repairs were also performed.

**Fig. (1):**
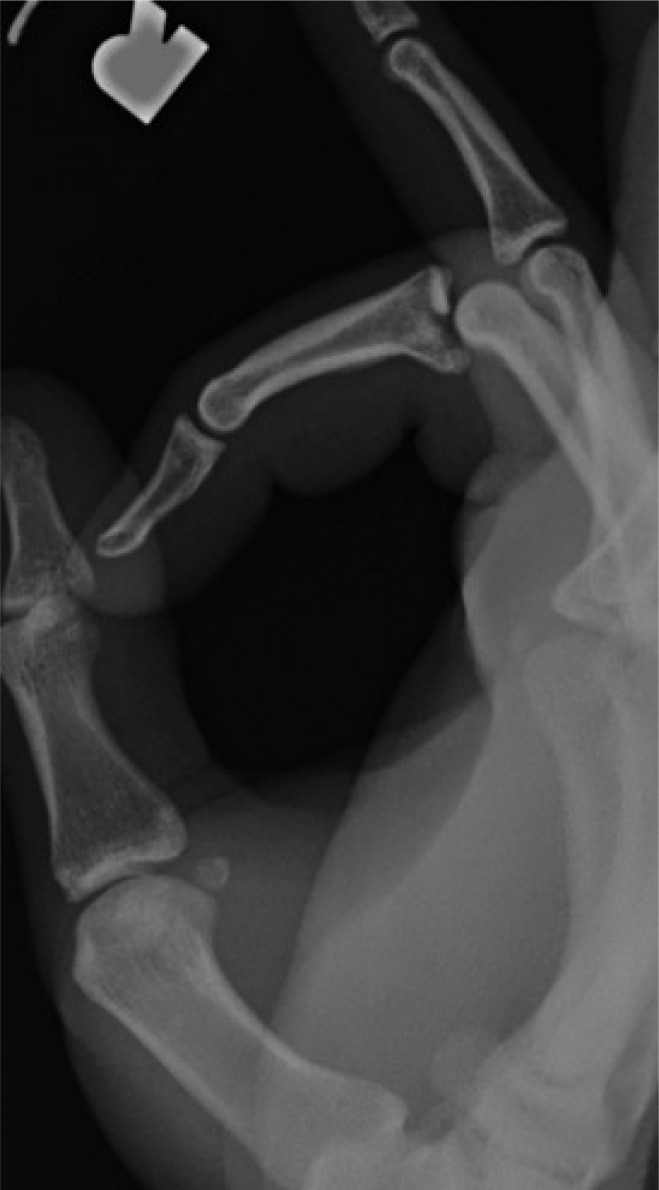
Patient 5 preoperative radiograph.

**Fig. (2):**
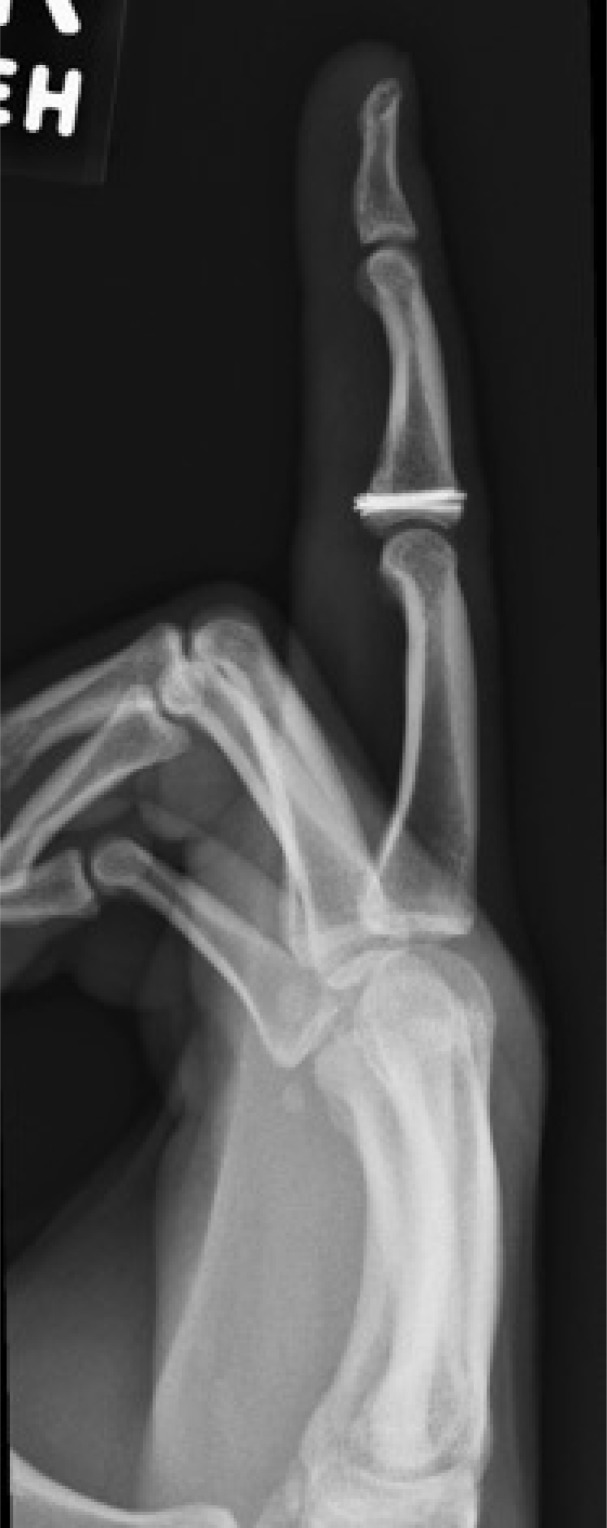
Patient 5 lateral radiograph at final follow-up.

**Fig. (3):**
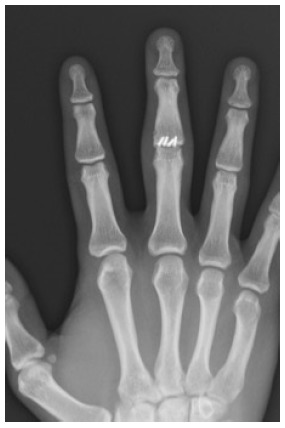
Patient 5 AP radiograph at final follow-up

**Fig. (4):**
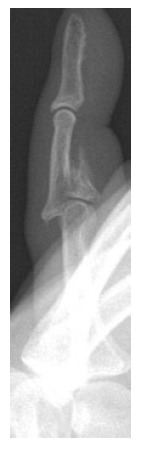
Patient 3 lateral preoperative radiograph.

**Fig. (5):**
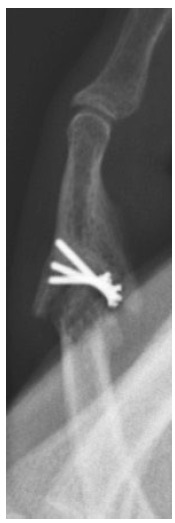
Patient 3 lateral radiograph at final follow-up.

Radiographic evidence of arthritis, range of motion, and subjective outcomes are shown in (Table **2**). (Figs. **1**-**3**) demonstrate a patient with restoration of the articular surface, while (Figs. **4**-**5**) show the development of post-traumatic arthritis in another patient.

### 3.1 Complications

1 patient was diagnosed with a surgical site infection and underwent irrigation and debridement 16 days after the index procedure and was placed on 1 week of oral antibiotics. The hardware was left in place and the infection resolved without any further intervention.

1 patient was found to have an atraumatic redislocation of the PIP joint 1 week after index procedure. This patient was taken to the operating room and underwent open reduction. The fracture alignment at the time of the secondary procedure was found to be intact and the hardware unchanged. The patient was closely followed post operatively and had no further subluxation of the joint.

## 4 DISCUSSION

The optimal treatment of comminuted PIP fracture dislocations is controversial. Several surgical options have been described including dorsal block pinning, external fixation, open reduction internal fixation. It is the philosophy of the authors that an attempt at anatomic reduction through a volar “shotgun” approach allowing to visualize the entire PIP articular surface is warranted even in highly-comminuted fracture dislocations.

Some authors believe that the volar approach is technically more demanding and feel that the extensive dissection of the volar tissues can lead to post-operative scarring and stiffness [4]. One long-term outcome study of 21 patients with basal fractures of the PIP joint demonstrated that comminuted fractures had the poorest subjective results that did not improve or worsen with time [5]. These authors observed a worse prognosis with comminuted and crushing injuries of the PIP and surgical intervention seldom improved results.

Ellis *et al.* evaluated 8 patients with PIP fracture dislocations treated with a dynamic external fixator [6]. At an average of 26 month follow-up, 5 of these patients were found to have evidence of post-traumatic arthritis, but minimal pain (0.6) based on a Visual analog scale from 0-10. Average PIP range of motion was 89 degrees in this cohort. Badia *et al.* also reported on the results of 6 patients who underwent simple dynamic external fixator [7]. Average PIP ROM was found to be 84 degrees and DIP ROM was 78 degrees.

Theoretical advantages of external fixation include distraction across the PIP joint, allow for healing without displacement force and fracture reduction through ligamentotaxis. It also allows for ROM beginning soon after application [8]. However, external fixation has narrow indications for this injury complex. Primarily, the fracture must be reducible with manipulation and must stay reduced through a full arc of motion. The results of this study showed an average arc of motion of 79° with a 60% incidence of post-traumatic arthritis. While formal comparison analysis was not performed with the small number of patients in each series, we believe the ROM after ORIF to be comparable to that of external fixation and is not subject to the same narrow indications required to perform external fixation successfully.

Dorsal block pinning is another minimally-invasive treatment option for the treatment of PIP fracture dislocations. Waris *et al.* published the results of 16 patients treated with extension block kirchner wire and a prebent intramedullary pin [9]. With a mean 5-year follow-up, average PIP ROM 83°, minimal pain as displayed with a value of 1 on VAS, and DASH scores of 4 out of 100 – indicating little functional impairment.

A number of studies have reported outcomes on the use of the volar approach and fixation of PIP fracture dislocations. Cheah *et al.* reported their results of ORIF with volar miniplate and screw fixation for dorsal fracture dislocation PIP joint with an average 25 month follow-up [10]. Of the 13 patients included in the study, 4 required hardware removal, 3 of which were performed as part of secondary procedure to improve range of motion. While all fractures went on to union, 3 went on to develop post-traumatic arthritis and 1 patient had joint redislocation. This study found an average arc of PIP motion to be 75° and DIP arc of motion to be 65°. In another case series, Hamilton *et al.* reported on 9 patients who underwent ORIF of unstable PIP fracture dislocations using the same volar approach [11]. With an average follow up of 42 months, the average arc of PIP motion was 70° and 3 of the 9 patients went on to develop degenerative joint disease.

Grant *et al.* reviewed 14 patients with fracture dislocations of the PIP joint with mini screw fixation using a volar approach [12]. They demonstrated that those injuries treated sub-acutely (over 14 days from time of injury) had decreased PIP ROM (86° vs 100°) and had a higher incidence of joint re-subluxation (21% vs 0%). Regardless, all patients were overall satisfied with their outcome based on general questionnaire.

Deitch *et al.* published a case series of patients treated with open reduction internal fixation by a variety of techniques and approaches including trans-articular k-wires to maintain reduction for 3 weeks and found an incidence of 12.5% redislocation [13]. This complication was attributed to cases with articular fragments involving over 50% of the articular surface.

In comparison to the results listed above, this study demonstrated an average PIP arc of motion of 79° degrees and a redislocation incidence of 20%. No hardware removal or manipulations were performed. Despite the development of arthritis in 60% of cases, the average MHQ result was quite high (95 of 100) indicating a highly functional PIP joint.

There are limitations to this study. This is a small retrospective case series and only 5 of 12 patients were successfully recruited to return to clinic for radiographic and clinical evaluation. With this small cohort, statistical comparison across studies was not feasible. However, despite small numbers we were able to achieve a mid-term follow-up with an average of 69 months after surgery and utilized a validated patient reported outcome tool in the Michigan Hand Questionnaire.

## CONCLUSION

Our findings would indicate that the volar approach to complex, comminuted PIP fracture dislocations treated with mini-screw internal fixation without supplemental fixation provides comparable objective outcomes to other treatment options for this injury pattern. As such, even amongst a number of newer techniques and fixation strategies, we believe this procedure remains a useful tool in the treatment armamentarium of these injuries. Despite a high incidence of post-traumatic arthritis, patients report excellent outcomes and no significant dysfunction with activities of daily living.

## Figures and Tables

**Table 1:** Patient demographics and preoperative radiographic findings.

Patient No.	Gender / Age (years)	Finger Involved	Mechanism of Injury	Time between Injury and Surgery (days)	Articular Involvement	# of Fracture Fragments
1	F/21	Left Small	Soccer Injury	17	>50%	>3
2	M/29	Left Middle	Circular Saw Injury	0	>50%	>3
3	F/64	Left Small	Ground-Level Fall	7	>50%	>3
4	M/56	Right Small	Bicycle Accident	5	50%	>3
5	M/25	Right Middle	Punched a Wall	7	>50%	>3

**Table 2:** Patient radiographic, objective, and subjective results.

Patient No	Finger Involved	Post-Operative Complications	Follow-up (months)	Radiographic Presence of Arthritis	VAS Pain Level (0-10)	MP ROM (°)	PIP ROM(°)	DIP ROM (°)	MHQ total score (Left)	MHQ total score (Right)	Overall ADL (Left)	Overall ADL (Right)
1	Left Small	Infection	33	None	0	90	100	90	100	87	100	100
2	Left Middle	None	81	Moderate	0	90	90	45	84	97	92	95
3	Left Small	Redislocation	40	Advanced	0	80	60	80	96	96	100	100
4	Right Small	None	133	Moderate	0	75	60	5	95	99	100	100
5	Right Middle	None	60	None	0	95	85	75	100	97	100	100

## LIST OF ABBREVIATIONS

CPT: = (Current Procedural Terminology)
DIP: = (Distal Inter-Phalangeal)
DASH: = Disabilities of the Arm, Shoulder, and Hand
MHQ: = (Michigan Hand Questionnaire)
ORIF: = (Open Reduction Internal Fixation)
PIP: = (Proximal Inter-Phalangeal)
ROM: = (Range of Motion)
VAS: = Visual Analog Scale
